# Organic Photothermal Cocrystals: Rational Design, Controlled Synthesis, and Advanced Application

**DOI:** 10.1002/advs.202206830

**Published:** 2023-01-27

**Authors:** Ye‐Tao Chen, Ming‐Peng Zhuo, Xinyi Wen, Wenbin Chen, Ke‐Qin Zhang, Ming‐De Li

**Affiliations:** ^1^ College of Chemistry and Chemical Engineering and Key Laboratory for Preparation and Application of Ordered Structural Materials of Guangdong Province Shantou University 515063 Shantou China; ^2^ National Engineering Laboratory for Modern Silk College of Textile and Clothing Engineering Soochow University Suzhou 215123 China; ^3^ Chemistry and Chemical Engineering Guangdong Laboratory Shantou University Shantou 515031 China

**Keywords:** charge transfer (CT) interaction, organic cocrystals, organic semiconductors, photoacoustic imaging, photothermal conversion

## Abstract

Organic photothermal cocrystals, integrating the advantages of intrinsic organic cocrystals and the fascinating photothermal conversion ability, hold attracted considerable interest in both basic science and practical applications, involving photoacoustic imaging, seawater desalination, and photothermal therapy, and so on. However, these organic photothermal cocrystals currently suffer individual cases discovered step by step, as well as the deep and systemic investigation in the corresponding photothermal conversion mechanisms is rarely carried out, suggesting a huge challenge for their further developments. Therefore, it is urgently necessary to investigate and explore the rational design and synthesis of high‐performance organic photothermal cocrystals for future applications. This review first and systematically summarizes the organic photothermal cocrystal in terms of molecular classification, the photothermal conversion mechanism, and their corresponding applications. The timely interpretation of the cocrystal photothermal effect will provide broad prospects for the purposeful fabrication of excellent organic photothermal cocrystals toward great efficiency, low cost, and multifunctionality.

## Graphical abstract

Organic cocrystal simply self‐assembled from two or more different chemical species through noncovalent interactions has great superiority over monocomponent on their unpredicted and regulated chemicophysical properties, which provides a valuable guidance for the development of photothermal conversion. This minireview highlights the recent advances of organic photothermal cocrystals on the rational design, controlled synthesis, and advanced application.

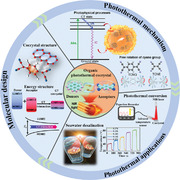

## 1 Introduction

Photothermic materials with fascinating light harvesting for thermal generation hold great promise toward fundamental importance and practical implication in actuators, micromotors, catalysis, and biomedicine.^[^ ^1^ ^]^ For example, Wu and co‐workers proposed an efficient catalytic system based on giant polyoxomolybdate and cationic cyclodextrin with desired near‐infrared (NIR) photothermal function.^[^ ^2^ ^]^ Compared with inorganic metal counterparts, organic semiconductors demonstrate significant progress in terms of photothermic applications, which contributes to their tailor‐made molecular structure, adjustable optical/electronic properties, low‐cost extensive preparation, and suitability for plastic substrates.^[^ ^3^ ^]^ Up to date, organic photothermic materials are mainly organic radicals,^[^ ^4^ ^]^ porphyrin,^[^ ^5^ ^]^ indocyanine green,^[^ ^6^ ^]^ polypyrrole,^[^ ^7^ ^]^ polymers or monomers,^[^ ^8^ ^]^ and polyaniline.^[^ ^9^ ^]^ A typical example is that Pu and co‐workers prepared a unique iron‐chelated semiconducting polycomplex nanoparticle, demonstrating significant photoacoustic imaging and photothermal abilities.^[^ ^10^ ^]^ However, these organic photothermic monomolecules commonly suffer sophisticated designs or complex preparation techniques, which have extremely restricted their practical applications.^[^ ^11^ ^]^ In order to meet the growing demand for photothermic applications, it is essential to develop a facile yet versatile solution route to rationally design and synthesize organic photothermic materials.

Organic cocrystals often exhibit superior or unpredicted chemicophysical properties in contrast with that of their monocomponent. Therefore, tremendous efforts have been concentrated on the fine preparation of the organic cocrystals comprising two or more different chemical species with an ordered molecular packing via a facile self‐assembly process.^[^ ^12^, ^13^ ^]^ For instance, the meso‐diphenyltetrathia‐annulene (DPTTA)‐C_60_ cocrystal presents great photoelectricity, which is absent in both pure DPTTA or C_60_.^[^ ^14^ ^]^ Significantly, organic charge‐transfer (CT) cocrystals exhibit the unique delocalization of electron cloud from the donor to the acceptor, which provides significant contributions to the orbital hybridization of both physicochemical properties and energy band engineering.^[^ ^15^ ^]^ Accordingly, organic CT cocrystals generally demonstrate plentifully desired optoelectronic features, including room‐temperature phosphorescent,^[^ ^16^ ^]^ room‐temperature ferroelectricity,^[^ ^17^ ^]^ thermal‐mechanical response,^[^ ^18^ ^]^ and ambipolar charge transport.^[^ ^19^ ^]^ Furthermore, the energy structure and optical properties of these organic CT cocrystals could be finely designed and tunable by intelligently selecting the suitable components and fine regulation of the intermolecular CT nature.^[^ ^20^ ^]^ Notably, the organic cocrystal strategy can effectively reduce the energy gap to achieve an obvious redshift in absorption and a high‐efficiency light‐harvesting capability,^[^ ^21^ ^]^ which processes a significant potential in photothermal conversion (PTC). Inspired by these advantages, organic PTC cocrystal is composed of two or more different organic molecules through noncovalent interactions and presents an enticing prospect to realize a broad absorption and tunable energy dissipation for the high‐performant photothermal convention, which attributes to enhance the light‐harvesting ability of cocrystals and increase rate of the corresponding nonradiative decay via quenching fluorescence.^[^ ^22^ ^]^ Owing to the unique *π*‐conjugated structure and the strong electron‐donating/withdrawing ability, tetrathiafulvalene (TTF), polycyclic aromatic hydrocarbons, tetracyanoquinones, or their derivates have been successfully applied to design and synthesize the organic CT cocrystals with excellent PTC.^[^ ^23^ ^]^ Furthermore, these organic photothermal cocrystals demonstrate great advantages in various applications, including photothermal imaging,^[^ ^24^ ^]^ photothermal therapy,^[^ ^25^ ^]^ and seawater desalination.^[^ ^26^ ^]^

Although the field of organic CT cocrystals was swiftly and violently developed recently, organic photothermal cocrystals are still in the stage of gradual discovery as well as the lack of systematic theoretical summary. Thus, the raw material screening of organic photothermal cocrystals misses the rational strategy, and ambiguous photothermal mechanism leads to a largely unsolved problem for future photothermal applications. This perspective of novel organic photothermal cocrystals introduces systematic summarization of recent advances from design principles to the structure–PTC performance relationship, which is helpful for the readers to understand this potential field and guide the development of novel PTC cocrystals. First, we introduce the factors that affect the performance of organic photothermal cocrystals, including light harvesting ability and nonradiative decay pathways. Remarkably, the CT interactions are essential for the capability of both light‐harvesting and nonradiative decay. CT interactions can help organic photothermal cocrystals to obtain a narrow energy gap via the adjustment of their energy level structure, which can broaden their absorption and facilitate the nonradiative decay, resulting in the effect of enhanced photothermal conversion being achieved. Therefore, the relationship between CT interactions and photothermal properties is fully discussed and summarized in this work. Then, we classify and summarize the photothermal cocrystals recently reported, including the raw material framework, photothermal performance, and PTC mechanism. Finally, we show the advanced and promising applications of these photothermal cocrystals, involving photothermal imaging, biological application, and seawater desalination.

## 2 Design and Synthesis of Organic CT Cocrystals with High‐Efficiency PTC

**Figure 1:**
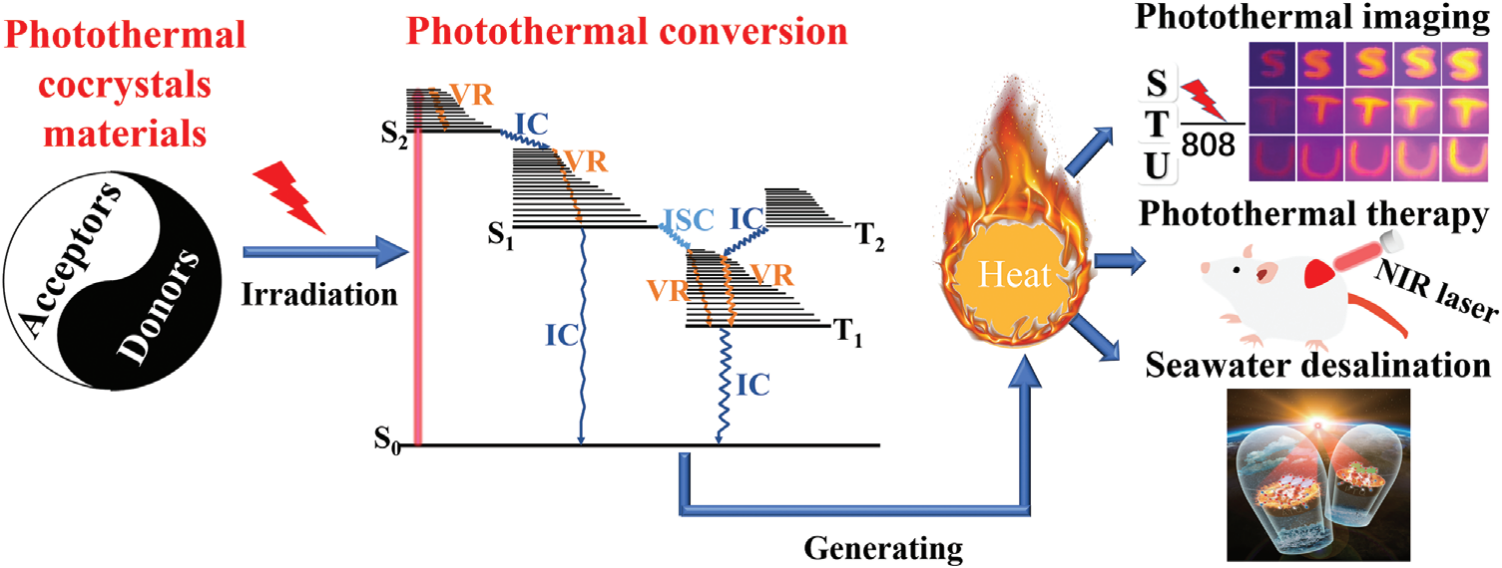
Schematic diagram of the mechanism and application of PTC cocrystal materials.

CT interactions are a powerful driving force that can be manipulated to assemble different single‐component structures into a double‐ or multicomponent structure in the solid state.^[^ ^27^ ^]^ Organic CT cocrystals were constructed by the donors and acceptors via the CT interaction to form a crystalline structure, which demonstrates superior photophysical properties to single components.^[^ ^28^ ^]^ Therefore, through effectively regulating the CT natures, including the degree of charge transfer, separation, recombination, charge generation, ground and excited state dynamics, and other processes, the desired functional cocrystals could be achieved. In addition, theoretical studies have shown that the delocalization of electrons transferred from their donor to their acceptor facilitates the adjustment of the CT cocrystal energy level structure, in which the highest occupied molecular orbital (HOMO) is more relevant to the donor HOMO, while the lowest unoccupied molecular orbital (LUMO) belongs mainly to the acceptor LUMO.^[^ ^29^ ^]^ Inspired by the orbital hybridization, through purposefully selecting donors and acceptors, cocrystal CT interactions can be effectively controlled, resulting in regulating its electron cloud density and energy level structure.^[^ ^30^ ^]^ Therefore, the cocrystal strategy allows for a controlled narrowing of the bandgap for red‐shifted emission/absorption^[^ ^31^ ^]^ and an effective increase in energy level density,^[^ ^32^ ^]^ thereby, increasing the absorption of light energy and conversion of heat energy,^[^ ^33^ ^]^ which provides a practical route for the rational design and synthesis of the desired photothermal material. For this review, the photothermal cocrystal materials were clarified, as well as a summary of photothermal cocrystal materials consisted of both their photothermal mechanisms and novel applications (**Figure** **1**). This perspective of novel organic photothermal cocrystals introduces systematical summarization of recent advances from design principles to the structure‐PTC performance relationship, which is helpful for the readers to understand this potential field and guide the development of novel PTC cocrystals.

We highlight the significant progress of the currently reported PTC cocrystal materials according to the differences in donor frames, such as the framework of TTF, the skeleton of *N,N,N,N*‐tetramethyl‐p‐phenylenediamine (TMPD), the framework of tetramethylbenzidine (TMB) and the frame of polycyclic aromatic hydrocarbons (**Table** **1**).

**Table 1:** Representative examples of PTC CT cocrystals

Molecular structure		Preparation method	Laser wavelength [nm]	Photothermic conversion efficiency [%]	Refs.
Donor	Acceptor				
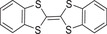	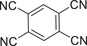	Slow evaporation	808	18.8	[44]
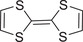	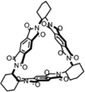	Slow evaporation	808	15.1	[45]
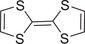	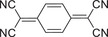	Slow evaporation		90.3	[46]
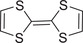	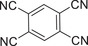	Slow evaporation	Solar irradiation	21.9	[52]
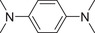	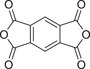	Drop‐casting	808	87.2	[48]
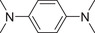	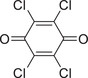	Solid‐phase grinding		–	[49]
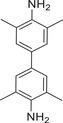	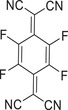	Nanoprecipitation	1064	42.4	[52]
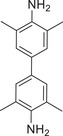	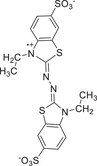	Coprecipitation	Solar irradiation	97	[53]
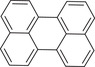	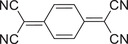	Reprecipitation	1064	42	[64]
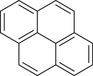	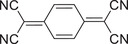	Slow evaporation	808	83.3	[72]
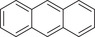	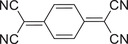	Slow evaporation	808	60.53	[73]
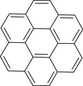	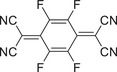	Solution self‐assembly	808	69.3	[74]
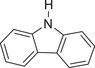	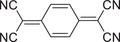	Slow evaporation	808	53.7	[76]
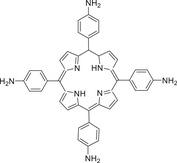	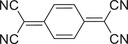	Reprecipitation	808	23.1	[77]

### 2.1 Photothermal Cocrystals Based on TTF Derivates

**Figure 2:**
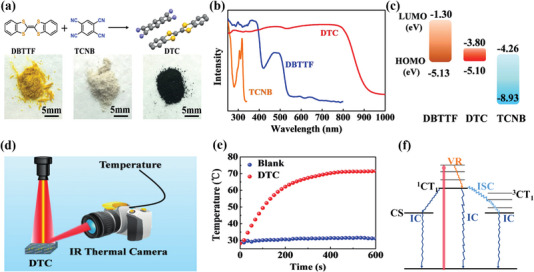
The photothermal‐conversion ability and mechanism of DBTTF‐TCNB cocrystal. a) Molecular structures and powders of DBTTF, TCNB, and DBTTF‐TCNB cocrystal. b) Absorption spectra of DBTTF, TCNB, and their cocrystal. c) Band diagram of DBTTF, DBTTF‐TCNB cocrystal, and TCNB. d) The device of the PTC measurement. e) PTC heating curves of DBTTF‐TCNB at 808 nm laser. f) Jablonski diagram of the DBTTF‐TCNB cocrystal. Reproduced with permission.^[^ ^24^ ^]^ Copyright 2018, Wiley‐VCH.

It is well known that the TTF molecule and the derivates exhibit a strong electron‐donating ability and great thermal stability,^[^ ^23a^ ^]^ which has been regarded as a promising building block to construct high‐performant photothermal cocrystal materials. Since the path breaking work by Hu and co‐workers, dibenzotetrathiafulvalene (DBTTF) and 1,2,4,5‐tetracyanobenzene (TCNB) have been employed as electronic donors and acceptors to form the typical D–A cocrystal (**Figure** **2**a).^[^ ^24^ ^]^ Owing to the strong CT interaction between the DBTTF and TCNB, the DBTTF‐TCNB cocrystal demonstrates an obvious red‐shift to obtain the desired NIR absorption (Figure 2b), in contrast with those of their monocomponents. It is agreed with the narrow HOMO–LUMO gap of 1.3 eV as shown in Figure 2c, which is beneficial for the efficient NIR PTC. Under 808 nm laser irradiation at a power density of 0.7 W cm^−2^, the temperature of the DBTTF‐TCNB powder rapidly elevated from room temperature to the highest state of 71.3 °C in 600 s, which was easily recorded by an IR thermal camera (Figure 2d,e). On the contrary, the blank sample without cocrystals rises less than 2.5 °C. The calculated PTC of DBTTF‐TCNB efficiencies is 18.8%, suggesting a high‐performance PTC. Notably, the Jablonski diagram of the DBTTF‐TCNB is proposed to reveal its PTC mechanism (Figure 2f). Under laser irradiation, the electron transitions from its ground state to the high‐energy excited state. Next, the electron will go back to its ground state via nonradiative pathways, including internal conversion (IC), charge‐separation (CS), and intersystem crossing (ISC), which is beneficial to its PTC process. Moreover, the ultrafast excited‐state relaxation kinetics is dominated by intensive CT interactions. This work offers novel perspectives for the advancement of PTC materials, as well as lays the foundation for better applications of photothermal materials. Inspired by this success, Zhao and co‐workers applied the pyromellitic TTF and diimide (Tri‐PMDI) to rationally design and synthesize a new type of organic CT cocrystal, which process a NIR PTC efficiency of 15.5% under 808 nm laser irradiation at a power density of 0.7 W cm^−2^ in 200 s.^[^ ^26^ ^]^ The large specific surface area and the dense mixed stacking structure of TTF‐Tri‐PMDI facilitate efficient light absorption and accelerate nonradiative pathways, respectively, which is conducive to the more efficient PTC process. In addition, its narrow HOMO–LUMO gap of 2.08 eV corresponds to a wavelength of 777 nm, consisting of the experimental absorption peak of 800 nm. Combing with the strong acceptor of 7,7,8,8‐tetracyanoquinodimethane (TCNQ), TTF was also applied to prepare a novel full solar PTC cocrystal of TTF‐TCNQ through a facile self‐assembly process.^[^ ^34^ ^]^ Based on a small energy gap of 0.44 eV and an over 2500 nm experimental absorption band, TTF‐TCNQ possessed a significant light‐collection capability in the range of 200–2500 nm, which demonstrated an efficient whole‐solar PTC, resulting in a great solar energy conversion efficiency of up to 90.3% under 1 sunlight.

### 2.2 Photothermal Cocrystals Based on Benzidine Derivants

**Figure 3:**
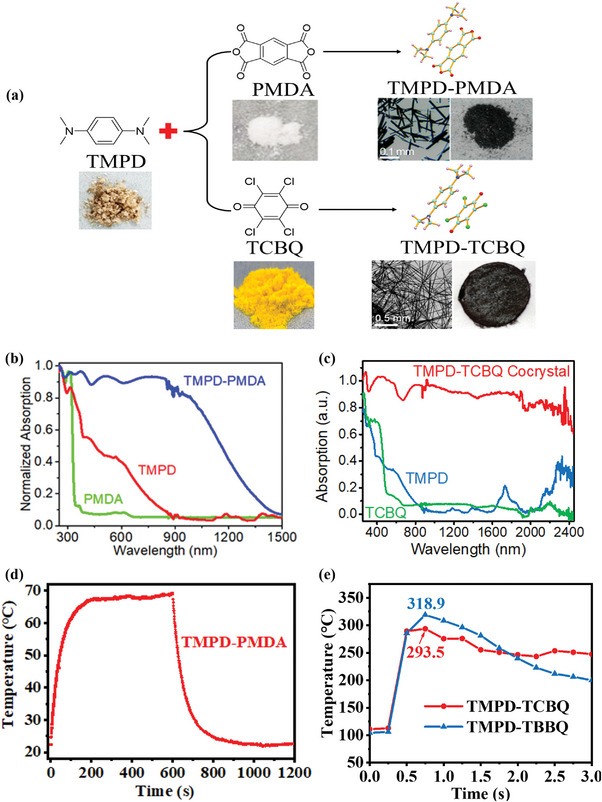
The photophysical properties of the PMDA‐TMPD and TMPD‐TCBQ cocrystals. a) Shown are the pictures and molecular structures of donors and acceptors, together with their cocrystals. b) Absorption spectra of PMDA, TMPD, and PMDA‐TMPD cocrystals. Reproduced with permission.^[^ ^36^ ^]^ Copyright 2021, American Chemical Society, c) TMPD, TCBQ, and TMPD‐TCBQ cocrystals. Reproduced with permission.^[^ ^37^ ^]^ Copyright 2022, American Chemical Society. d) The heating and cooling curves of TMPD‐PMDA at 808 nm. e) PTC curve of TMPD‐TCBQ at 808 nm.

The electron‐rich planar structure of TMPD is widely applied as an electron donor to induce an enhancing CT integration with the electron‐withdrawing acceptors for forming the organic CT cocrystals.^[^ ^35^ ^]^ Owing to the intense CT interaction, the TMPD‐based cocrystal materials generally exhibit a narrow energy gap with a broad UV–Vis–NIR absorption, showing a wonderful potential for photothermal applications. Considering these advantages, our group successfully employed the typical electron acceptor of pyromellitic dianhydride (PMDA) and tetrachloro‐1,4‐benzoquinone (TCBQ) to rationally design and synthesize two kinds of TMPD‐based cocrystal materials of TMPD‐PMDA^[^ ^36^ ^]^ and TMPD‐TCBQ^[^ ^37^ ^]^ as illustrated in **Figure** **3**a. Both TMPD‐PMDA and TMPD‐TCBQ have a relatively broad absorption band accompanied by a substantial absorption value in the NIR region (Figure 3b,c), which displays a promising potential for PTC. Remarkably, the temperature of the TMPD‐PMDA could increase rapidly from room temperature to 70 °C in 200 s under the irradiation of a 0.65 W 808 nm laser (Figure 3d), suggesting an excellent PTC performance. Due to the effective nonradiative decay, the corresponding PTC efficiency is up to 87.2%, which is the highest among the organic PTC cocrystal materials reported. Under the irradiation of an 808 nm laser, the electron will rapidly transition from the ground CT state to the excited CT state, then return to the ground state via rapid nonradiative decay. The main nonradiative decay process includes a high rate (94.4%) of vibrational relaxation (VR) and IC processes with ultrafast excited‐state decay (0.12 ps), which are beneficial to the efficient PTC. Similarly, the TMPD‐TCBQ cocrystal could expand and release a large amount of heat when its temperature reaches a specific critical value under NIR laser irradiation, resulting in a rapid rise in the temperature of the cocrystal system to 293.5 °C. Furthermore, cocrystals obtained after the replacement of its acceptor has a post‐expansion temperature of up to 318.9 °C. Remarkably, its excellent PTC ability is attributed to a dramatic exothermic thermal polymerization reaction (Figure 3e).

**Figure 4:**
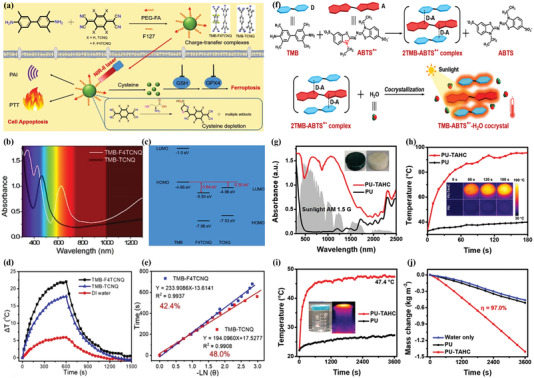
Illustration of the self‐assembly for the tumor‐targeted CT complex nanoparticles and the CT complex with sandwich structure. a) Studying on the preparation of tumor‐targeted CT complex nanoparticles and diagram of ferroptosis and NIR‐II triggered Phototherapy. b) UV–vis–NIR absorption spectra of TMB‐TCNQ and TMB‐F_4_TCNQ. c) Calculated energy bands of TCNQ or F_4_TCNQ, TMB, and their corresponding cocrystals. d) The heating and cooling curves of TMB‐TCNQ and TMB‐F_4_TCNQ NPs and DI water at 1060 nm. e) Plot of time versus‐LN(*θ*) of TMB‐TCNQ and TMB‐F_4_TCNQ NPs, *θ* is the driving force of temperature. Reproduced with permission.^[^ ^25^ ^]^ Copyright 2021, Wiley‐VCH. f) Diagram of CT complex formation based on the sandwich structure as well as the crystallization of TMB‐ABTS^+•^(TAHC) and its corresponding process of sunlight‐thermal conversion. g) Absorption spectra of PU and PU‐TAHC foams. h) Temperature changes of PU and PU‐TAHC foams under irradiation in air and the corresponding thermal images. i) Temperature changes of two foams floating on water against irradiation time. j) Water evaporation curves of two foams. Reproduced with permission.^[^ ^42^ ^]^ Copyright 2022, Wiley‐VCH.

On the other hand, a stable CT complex based on the intense *π*–*π* interaction between TMB and oxidized TMB demonstrates the intense NIR‐II (1064 nm) in situ photoacoustic signal and biodegradable,^[^ ^38^ ^]^ which processes an immense potential in NIR photothermal applications.^[^ ^39^ ^]^ Notably, it has been widely applied for redox‐activatable NIR photoacoustic imaging and photothermal therapy. Dong and co‐workers prepared a D–A cocrystal based on 7,7,8,8‐tetracyanoquinonedimethane derivates (F*_x_* TCNQ) and TMB, which has a NIR‐II induced photothermal property (**Figure** **4**a).^[^ ^25^ ^]^ Significantly, both TMB‐TCNQ and TMB‐F_4_TCNQ cocrystals possess intense intermolecular CT interaction, which would promote the space charge transfer from TMB to acceptors of TCNQ or F_4_TCNQ. Moreover, TMB‐TCNQ and TMB‐F_4_TCNQ demonstrated a broad absorption from the UV to NIR‐II region (Figure 4b). Furthermore, the narrower energy gap of TMB‐TCNQ and TMB‐F_4_TCNQ is 0.30 eV and 0.84 eV, respectively (Figure 4c), which were conducive to the nonradiative process of these cocrystals for a great NIR photothermal ability.^[^ ^40^ ^]^ Under 1060 nm laser irradiation, the PTC efficiency of TMB‐TCNQ and TMB‐F_4_TCNQ were 48.0% and 42.4%, respectively (Figure 4d,e), suggesting a great potential in NIR‐II photothermal application. Furthermore, the electron donor of TMB could self‐assembly into other CT cocrystals with a mixed packing mode via combining with an electron acceptor of 2,2’‐azino‐bis‐(3‐ethylbenzothiazoline‐6‐sulfonic acid) radical cations (ABTS^+•^).^[^ ^41^ ^]^ The sandwich structure of the TMB‐ABTS^+•^ cocrystal contributes to the electron transfer from TMB with a substantial electron delocalization to the ABTS^+•^ with a high electron affinity(Figure 4f).^[^ ^42^ ^]^ Moreover, the intrinsic broad absorption of ABTS^+•^ further promotes the light absorption of the TMB‐ABTS^+•^ in the entire solar spectrum (Figure 4g,h). Therefore, TMB‐ABTS^+•^ demonstrated a high solar‐to‐vapor efficiency of 97.0% (Figure 4i,j), which revealed an ability of excellent solar‐thermal conversion.

### 2.3 Photothermal Cocrystals Based on Polycyclic Aromatic Hydrocarbons

**Figure 5:**
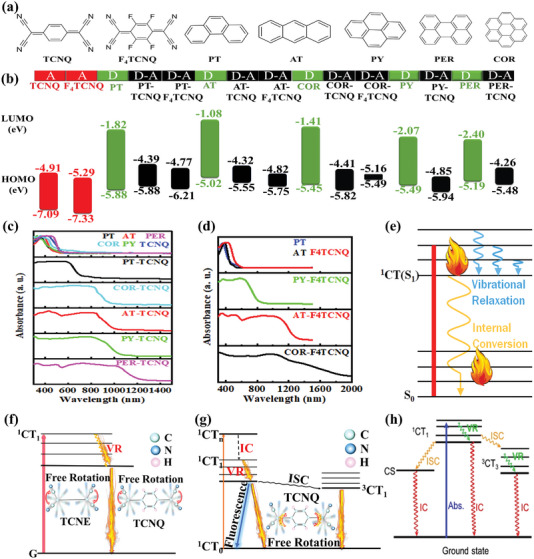
The photophysical characterizations and photothermal‐conversion mechanisms of cocrystals based on polycyclic aromatic hydrocarbons. a) Molecular structures and b) calculated energy bands of TCNQ or F4TCNQ, polycyclic aromatic hydrocarbon, and their corresponding cocrystals (D: donor; A: acceptor) (Basic set: COR‐TCNQ, and PER‐TCNQ(B3LYP/6‐31G(d,p)); PY‐TCNQ(HSEH1PBE/6‐311+G(d,p));PT‐TCNQ, AT‐TCNQ, PT‐F4TCNQ, and AT‐F4TCNQ(BLYP IOp (3/76 = 1000001300), IOp (3/77 = 072000 8700), IOp (3/78 = 0810010000)/6‐311G (d,p)); COR‐F4TCNQ(B3LYP‐D/6‐31G(d,p))). UV–vis–NIR absorption spectra of c) TCNQ or d) F4TCNQ, polycyclic aromatic hydrocarbon, and their corresponding cocrystals. e) Jablonski diagram of excited‐state evolution for COR‐TCNQ, and PER‐TCNQ. Reproduced with permission.^[^ ^55^ ^]^ Copyright 2021, Wiley‐VCH. f) PY‐TCNQ. Reproduced with permission.^[^ ^56^ ^]^ Copyright 2021, American Chemical Society. g) PT‐TCNQ, AT‐TCNQ, PT‐F4TCNQ, and AT‐F4TCNQ. Reproduced with permission.^[^ ^57^ ^]^ Copyright 2022, American Chemical Society. h) COR‐F4TCNQ cocrystals. Reproduced with permission.^[^ ^58^ ^]^ Copyright 2022, American Chemical Society.

Owing to the unique *π*‐conjugated molecular structure,^[^ ^23b‐d^ ^]^ polycyclic aromatic hydrocarbons (PAHs) have generated immense scientific interest in the optoelectronic applications, such as organic solar cells,^[^ ^43^ ^]^ organic light‐emitting diodes (OLEDs),^[^ ^44^ ^]^ organic solid‐state lasers (OSSLs),^[^ ^45^ ^]^ and organic field‐effect transistors (OFETs).^[^ ^46^ ^]^ Furthermore, the PAHs also exhibit a strong electron‐donating ability, rendering them promising electronic donors for rational design and synthesis of novel organic cocrystals with desired optoelectronic properties, including bipolar transport of electrons,^[^ ^47^ ^]^ tunable emission color, PTC, two‐photon absorption,^[^ ^48^ ^]^ and ferroelectric properties.^[^ ^49^ ^]^ Owing to the strong CT interaction, the PAHs‐based CT cocrystals generally have a narrowed energy bandgap,^[^ ^50^ ^]^ which is beneficial for achieving an effectively quenching fluorescence for the high‐performant photothermal functions. Impressively, both the immense electron‐withdrawing effect and the planar molecular packing promote the presence of high electron mobility for F*_x_* TCNQ in the organic CT cocrystals,^[^ ^23e^ ^]^ which has great potential in OFETs. Moreover, due to the great ambipolar transport properties, F*_x_* TCNQ is also regarded as electronic acceptors for innovative organic cocrystals, which has significant research value and application prospects in organic optoelectronics, such as luminescence,^[^ ^51^ ^]^ charge transport,^[^ ^52^ ^]^ and photoresponse.^[^ ^53^ ^]^ Significantly, the effective separation and recombination of electrons and holes in F*_x_* TCNQ could induce a narrow bandgap,^[^ ^54^ ^]^ which is a candidate acceptor for forming the high‐performant PTC cocrystal. Recently, PAHs and F*_x_* TCNQ were successfully employed in organic CT cocrystals for the application of PTC. Lee and co‐workers prepared CT cocrystal nanoparticles (NPs) based on perylene (PER) and TCNQ, which possess a NIR‐II induced photothermal property.^[^ ^55^ ^]^ Remarkably, the calculated narrow energy gap of the PER‐TCNQ is only 1.22 eV (**Figure** **5**a,b), indicating that its light‐harvesting ability will be increased in the NIR‐II region and results in ultrafast excited‐state relaxation. Compared with the narrow absorption spectra of the pure PER and TCNQ without absorption in the NIR region, the PER‐TCNQ cocrystal demonstrates significant red‐shifted absorptions with peaks around 1040 nm, as shown in Figure 5c. The corresponding redshift spectrum to the NIR‐II regions in the experiment is consistent with its bandgap calculation. Attributing to its immense NIR‐II absorption, the temperature of the PER‐TCNQ NPs solution sharply increased to 34 °C under the irradiation of a 1064 nm laser, resulting in a calculated NIR PTC efficiency of 42%. Under the excitation of a 1064 nm laser, the electron transitions from its ground CT (S_0_) state to the high energy excited state (S_1_). Simultaneously, it will be back to its ground state through the processes of both VR and IC, which can generate plenty of heat in these processes. Therefore, the PTC ability of PER‐TCNQ is mainly attributed to a high rate of nonradiative decay via the process of VR and IC (Figure 5e).

To further reveal the generated heat process in VR and IC, our group designed a special cocrystal with the property of the free rotation of the —C(CN)_2_ group based on pyrene (PY) and TCNQ with the —C(C≡N)_2_ group, which is the determining way to stimulate the efficient nonradiation transition of excited charge transfer state, so as to achieve excellent PTC ability.^[^ ^56^ ^]^ Significantly, the infrared (IR) vibrational spectra of PY‐TCNQ demonstrated the characteristic peak of the C≡N stretching vibration with high blue shifts from 2228 cm^−1^ (TCNQ) to 2215 cm^−1^ (PQC), indicating an intense CT interaction between the PY and TCNQ. Beneficial from the strong CT interaction, PY‐TCNQ cocrystal displayed a significant red shift in the absorption spectrum of the experiment, obtaining a desired NIR absorption (Figure 5c), which is agreed with its narrow HOMO–LUMO gap of 1.09 eV (Figure 5a,b), suggesting that it is beneficial for the efficient NIR PTC. Therefore, under NIR laser irradiation, the temperature of PY‐TCNQ could rapidly increase from room temperature to 60 °C under 1.0 W 808 laser irradiation, resulting in excellent PTC efficiency of 83.3%. Remarkably, its PTC ability is derived from its photophysical process of exciton decay after laser irradiation, as shown in Figure 5f. Specifically, when PY‐TCNQ was irradiated by 808 nm laser, the electron would transit from the ground state to the singlet charge‐transfer state (^1^CT_1_). Next, the electron returned to the ground state through the VR and IC processes in turn. Interestingly, the —C(C≡N)_2_ group of freely rotating is in favor of generating heat after infrared excitation, opening a channel for effective nonradiative decay through the VR and IC. In conclusion, the free rotation of the —C(C≡N)_2_ group of PY‐TCNQ can provide more chances for nonradiative transition to generate substantial heat.

Inspired by these works, our group further explored the effect of regulating the rotation of the —C(C≡N)_2_ group. Interestingly, we found that intermolecular interactions of *π*–*π* stacking and p–*π* interactions could affect the rotation of —C(C≡N)_2_ groups.^[^ ^57^ ^]^ To verify this speculation, we selected TCNQ as the acceptor, combining it with the electronic donors of phenanthrene (PT) and anthracene (AT), respectively, which obtained the PT‐TCNQ and AT‐TCNQ cocrystals. Both PT‐TCNQ and AT‐TCNQ have an obvious red‐shifted absorption spectrum (Figure 5c), while AT‐TCNQ possesses an extensive absorption value in the NIR region. Therefore, the ability of the NIR PTC of AT‐TCNQ is stronger than that of PT‐TCNQ, and their NIR PTC efficiencies are 60.53% and 35.85%, respectively. Interestingly, their various PTC abilities originate from the different intermolecular interactions between donors and acceptors, resulting in diverse regulation of the spin of the —C(C≡N)_2_ group. The specific photophysical processes of the excited state of PT‐TCNQ and AT‐TCNQ cocrystals are shown in Figure 5g. First, the electrons can transfer from the ground state (CT_0_) to the high‐energy singlet CT*_n_* state under NIR irradiation. Then, the excited state will undergo a nonradiative transition of VR, IC, and ISC, eventually returning to the ground state via competitive radiation. Remarkably, the —C(C≡N)_2_ group of freely rotating is beneficial to generating heat during nonradiative decay processes of VR, IC, and ISC. Therefore, the ability of NIR PTC of AT‐TCNQ is different from that of PT‐TCNQ, which originates from the effect of the —C(C≡N)_2_ group of freely rotating through regulating by the intermolecular interaction. Specifically, the intense *π*–*π* interaction is in favor of gaining a broad absorption spectrum, inducing an increased light‐harvesting ability at NIR, which promotes the conversion of more NIR light energy into heat via the —C(C≡N)_2_ group of freely rotating. In contrast, p–*π* interaction can inhibit the rotation of —C(C≡N)_2_ groups, restraining the nonradiative decay of the excited state. Therefore, the PTC efficiency of AT‐TCNQ is higher than that of PT‐TCNQ, which is consistent with the stronger *π*–*π* interaction and weaker p–*π* interaction of AT‐TCNQ. It is illuminated that the intense *π*–*π* interaction and weak p–*π* interaction are in favor of nonradiative decay through the —C(C≡N)_2_ group of freely rotating, greatly promoting PTC performance of cocrystals.

Besides, Zhuo and co‐workers successfully prepared a great organic photothermal cocrystal nanowire based on coronene (COR) and 2,3,5,6‐tetrafluoro‐7,7,8,8‐tetracyanoquinodimethane (F_4_TCNQ) via the bottom‐up approach, further integrating cocrystal material into the polyurethane (PU) for the formation of the nanofiber membrane through electrospinning technology.^[^ ^58^ ^]^ Interestingly, COR‐F_4_TCNQ cocrystal with an intense electron delocalization possesses a narrow energy gap of 0.33 eV (Figure 5a,b), suggesting a desired NIR absorption. As expected, COR‐F_4_TCNQ demonstrates a wide absorption from 250 to 1800 nm (Figure 5d), which is beneficial for promoting the NIR PTC. Therefore, under 808 nm laser irradiation, COR‐F_4_TCNQ displays a rapidly increased temperature from 22.4 to 82 °C in 15 s, indicating a high PTC efficiency of 62.2%. Furthermore, COR‐F_4_TCNQ demonstrates its photophysical processes of the excited state via the Jablonski diagram, as shown in Figure 5h. Specifically, when excited, the electrons can transfer from the CT ground state to the CT excited state (^1^CT_1_). Next, the electrons will go through the nonradiative pathways of VR, ISC, and IC, finally returning to the ground state. Notably, these nonradiative pathways are favorable for the high‐efficiency PTC, which attributes to the intense CT interactions and its unique molecular orbitals. On the one hand, the decay kinetics of rapidly excited CT states is mainly controlled by intense CT interactions.^[^ ^59^ ^]^ Mixed stacking arrays of COR‐F_4_TCNQ could enhance the effect between intermolecular interactions and the delocalization of *π* electrons, hindering its emission process, which promotes more dissipation of excited state energy in nonradiative mode. On the other hand, due to COR‐F_4_TCNQ with novel molecular orbitals, excitons are generated in the excited state, which inhibits the green‐emission optical transition of pure coronene. Based on these two aspects, electrons of COR‐F_4_TCNQ can effectively occur the nonradiative decay via VR, IC, and ISC after NIR laser irradiation, which significantly improves the PTC ability of COR‐F_4_TCNQ. Remarkably, although there are few errors in the comparison of energy gap and absorption in different reports, these photothermal cocrystals possess the common characteristics of narrow energy gap and broad absorption spectra.

Meanwhile, Chen and co‐workers reported a D–A cocrystal based on carbazole (CZ) derivates and TCNQ, which process a NIR PTC property for photothermal imaging and functional electrical device control.^[^ ^60^ ^]^ Furthermore, Xie and co‐workers prepared a CT cocrystal of nanoscale assemblies based on 5,10,15,20‐tetrakis(4‐aminophenyl) porphyrin (TAPP) and TCNQ, further applying in both photodynamic and photothermal therapy.^[^ ^61^ ^]^

In conclusion, the excellent PTC properties affected by the molecular configurations could be concluded into three points. First, matching molecular configuration could increase the CT interaction of the cocrystal, further broadening the absorption band and reducing the energy bandgap. It will facilitate the enhanced light‐harvesting ability of cocrystals, as well as the increased rate of the corresponding nonradiative decay. Secondly, based on the strong CT interaction, the molecular configuration could effectively occur quenching fluorescence for the high‐performant photothermal functions. Thirdly, special molecular configurations could provide a tailormade channel for nonradiative transitions, further facilitating the process of utilizing photo energy to convert thermal energy, which obtains high‐performant photothermal cocrystal. In a word, we can summarize that PTC process of organic cocrystal is mainly attributed to the nonradiative process of VR, IC, and ISC (Figure 1). However, due to the antagonism between the radiative and nonradiative decay pathways, we briefly introduce the design strategy of cocrystal‐based luminescence agents to help readers easily realize the design of PTC cocrystals. Specifically, the regulation of the luminescence properties of cocrystals mainly originates from intermolecular interactions, including hydrogen bonds, and halogen bonds and so on.^[^ ^62a,b^ ^]^ For example, Hu and co‐workers have reported pyrene‐octafluoronaphthalene (pyrene‐OFN) and pyrene‐1,2,4,5‐tetracyanobezene (pyrene‐TCNB) cocrystals that they possess distinct luminescence properties, which attribute to their different intermolecular interaction modes.^[^ ^62c^ ^]^ Furthermore, Hu and co‐workers proposed a cocrystal of persistent luminescence with an room‐temperature phosphorescent lifetime as high as 2 s, which originates from the intensive hydrogen bonds interactions for enhancing the efficiency of activated delayed fluorescence (TADF)‐assisted energy transfer route.^[^ ^62d^ ^]^

## 3 Applications of Organic Photothermal Cocrystals

Organic PTC materials with the properties of fascinating light‐harvesting for thermal generation have attracted much attention in the past few decades due to their potential applications in wearable devices, solar photothermal electrodes, smart hydrogels, and other fields.^[^ ^63^ ^]^ Therefore, it is urgent to regulate and control the PTC behavior of organic solid materials to obtain high‐performance PTC efficiency. Organic cocrystals consist of noncovalent bonds, which are relatively easier to regulate than covalent bonds.^[^ ^31b^ ^]^ Furthermore, they are composed of two or more components. The introduction of additional molecules can reduce the bandgap of the material, resulting in increasing the light‐harvesting capability and the rate of nonradiative transitions,^[^ ^32^ ^]^ which may help to improve the PTC efficiency of the cocrystal with respect to the constituent units. Within this section, we will explore the potential of the photothermal cocrystal materials for photothermal imaging, biological application, seawater desalination, and some innovative applications, which will provide novel insight into future photothermal applications.

### 3.1 Photothermal Imaging

**Figure 6:**
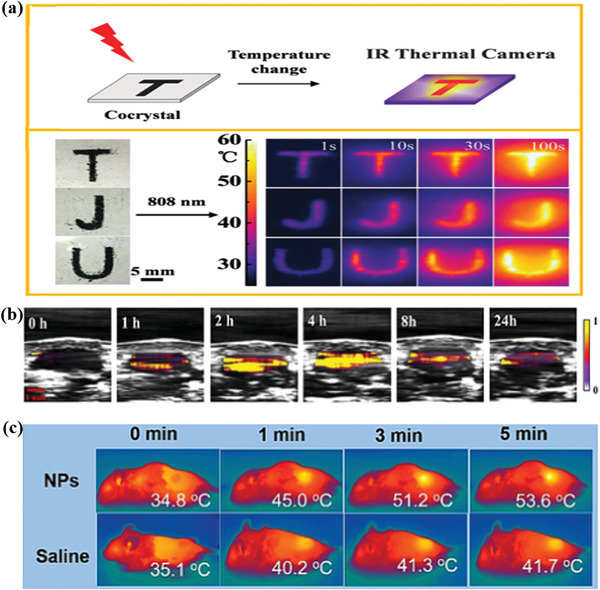
The process of photothermal imaging and photoacoustic imaging based on photothermal cocrystals. a) Photothermal process imaging diagram of cocrystal under 808 nm laser. Reproduced with permission.^[^ ^24^ ^]^ Copyright 2018, Wiley‐VCH. b) Pictures of photoacoustic imaging of tumor‐bearing mice at various time after tail‐intravenous injection excited by 1300 nm laser. c) Infrared temperature change images of tumor‐bearing mice injected with TMB‐F4TCNQ NPs or saline as the irradiation time at 1060 nm laser (*P* = 1.0 W cm^−2^). Reproduced with permission.^[^ ^25^ ^]^ Copyright 2021, Wiley‐VCH.

Photothermal imaging is a technique in which materials simultaneously or step by step use light and heat energy to record the imaging process.^[^ ^64^ ^]^ Compared with traditional optical imaging, photothermal imaging is a noninvasive technique with images of higher resolution and deeper perforation. To date, common photothermal imaging materials are mainly noble metals,^[^ ^65^ ^]^ polymers such as polypyrrole,^[^ ^66^ ^]^ porphysome,^[^ ^67^ ^]^ and boron‐dipyrromethene dye.^[^ ^68^ ^]^ However, both the high cost and complex synthesis have limited the development of photothermal imaging fields. Due to the low cost and flexible synthesis of cocrystal strategy, it has a broad prospect in the rational design and facile preparation of photothermal imaging functional materials. Since the pioneering work by Hu and co‐workers, the DBTTF‐TCNB cocrystal was first applied in photothermal imaging.^[^ ^24^ ^]^ Under the irradiation of 808 nm laser, the TJU pattern fabricated with cocrystal became great relucent in a short time (**Figure** **6**a), as well as the corresponding temperature imaging of the TJU pattern, was taken at the start of NIR light irradiation. Under the temperature imaging observation, the TJU pattern gradually brightens with the NIR light irradiation, indicating the cocrystal material with a high‐quality imaging effect. To further promote the effect of biological diagnosis, Dong and co‐workers further used the TMB‐F_4_TCNQ cocrystal with a photothermal imaging property to photoacoustic imaging (PAI).^[^ ^25^ ^]^ After tumor location in mice was injected with medicine and TMB‐F_4_TCNQ cocrystal materials, the photoacoustic signal increased gradually and reached the peak after injection for 4 h (Figure 6b), suggesting an active and passive targeting effect on solid tumors. Furthermore, the photothermal influence of TMB‐F_4_TCNQ was investigated in vivo after injection for 4 hours at 1060 nm (Figure 6c). Within 5 min, the temperature increased rapidly from 34.8 °C to 53.6 °C, reaching a temperature that could kill cancer cells, while the temperature of the saline group was only about 6.6 °C. These results elucidate that TMB‐F_4_TCNQ cocrystal is able to efficiently aggregate at tumor sites through passive and active targeting and demonstrate great NIR‐II‐triggering photoacoustic imaging ability in vivo. In a word, cocrystal material for photothermal imaging significantly broadens its application in multidisciplinary fields, in particular biomedicine.

### 3.2 Biological Application

It is well known that NIR light has the advantage of deeper tissue penetration and less biological interference.^[^ ^69^ ^]^ Therefore, NIR PTC materials have irreplaceable advantages in photothermal therapy. Due to the low absorption in the NIR wavelength range of biological cells and tissues, NIR light has the advantage of being less damaging to tissues.^[^ ^70^ ^]^ To date, NIR light has been widely used in biomedicine, particularly in photothermal therapy^[^ ^71^ ^]^ and photothermal antibacterial therapy.^[^ ^72^ ^]^ For example, NIR photothermal therapy mainly involves injecting photothermal materials into the tumor and then using a NIR laser to irradiate the tumor area at a fixed point so as to achieve the selective resection of the tumor.^[^ ^73^ ^]^ Furthermore, the photothermal antibacterial is mainly to inoculate the photothermal material to the wound infected by bacteria and then use NIR light for fixed‐point irradiation, resulting in achieving the effect of treating wound bacteria infection.^[^ ^55^ ^]^ At present, the traditional organic PTC materials, including polymer polypyrrole,^[^ ^74^ ^]^ polyaniline,^[^ ^75^ ^]^ and organic small molecule dyes,^[^ ^76^ ^]^ are successfully used in the biomedical field. However, to achieve a better effect of NIR photothermal treatment, traditional organic PTC materials need to obtain substantial value in the NIR region via complicated design and tedious synthesis, which greatly limits their practical applications. To meet the increasing biomedicine demands for organic PTC materials, a cocrystal strategy with flexible design and simple preparation was introduced into the excellent NIR PTC materials.

**Figure 7:**
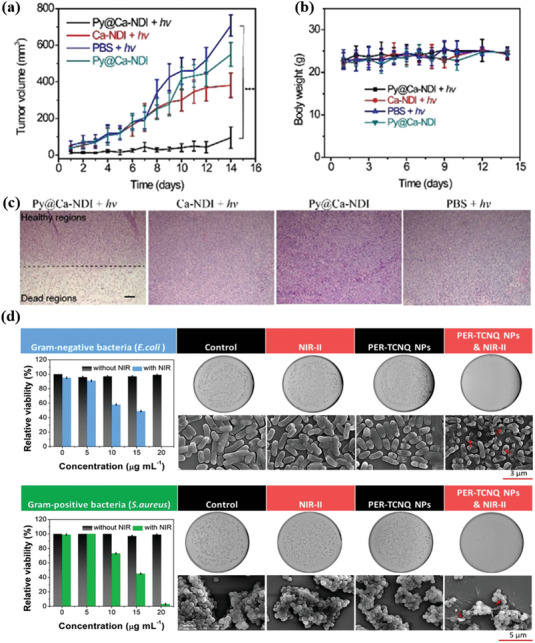
Illustration of the photothermal cocrystals for photothermal therapy. a) Tumor growth curves of 4T1 tumor‐bearing mice after various therapies. b) Body weight changes in 4T1 tumor‐bearing mice under various therapies. c) Images of Histological H&E analysis for tumor tissues in each group of mice after therapies. Reproduced with permission.^[^ ^77^ ^]^ Copyright 2021, Wiley‐VCH. d) Inhibitory activity of PER‐TCNQ nanoparticles (0, 5, 10, 15, 20 mg mL^−1^) and their SEM images against *Escherichia coli* and *Staphylococcus aureus* after treatment by PER‐TCNQ nanoparticles (20 mg mL^−1^) with 1064 nm laser (1 W cm^−2^) irradiation and without irradiation. Reproduced with permission.^[^ ^55^ ^]^ Copyright 2021, Wiley‐VCH.

Recently, several significant works of organic cocrystal materials have been applied in cancer photothermal treatment and antibacterial therapy. Han and co‐workers first reported a host–guest MOF Py@Ca‐NDI cocrystal based on a metal‐organic framework (MOF) and PY for photothermal cancer treatment in vivo.^[^ ^77^ ^]^ Notably, the electron‐deficient MOF was constructed by a ligand of naphthalene diamide (NDI), and the metal nodes of Ca^2+^. The prepared Py@Ca‐NDI aqueous solution was used to eliminate the 4T1 tumor‐bearing in mice via photothermal treatment. The mice of 4T1 bearing were divided into four various treatment groups: 1) PBS + NIR irradiation; 2) Py@Ca‐NDI; 3) Ca‐NDI + NIR irradiation; 4) Py@Ca‐NDI + NIR irradiation. With treatment for 14 d, the volume of the 4T1 tumor‐bearing significantly reduced in group 4 (Py@Ca‐NDI + NIR light), as verified in **Figure** **7**a. It is powerfully confirmed that the growth of the 4T1 tumor‐bearing growth was notably restrained. After treatment in the Py@Ca‐NDI + NIR light group, histologic hematoxylin and eosin (H&E) analysis of tumor tissue revealed a distinct boundary between normal and severe necrosis (Figure 7c). It clarifies that the Py@Ca‐NDI cocrystal under NIR light could effectively eliminate the tumor tissue. Moreover, all groups of mice exhibited similar trends in body weight, but all slowly increased, reflecting that these treatments do not have a systemic toxic effect on the mice (Figure 7b). It indicates that the host‐guest MOF, Py@Ca‐NDI demonstrated an excellent photothermal effect for cancer treatment. Similarly, Dong and co‐workers prepared a multifunctional nanococrystal material through supramolecular assembly and nanoprecipitation for cancer photothermal treatment.^[^ ^40^ ^]^ Strikingly, the mechanism of inhibiting the growth rate of tumors is that TMB‐F_4_TCNQ could be selectively and efficiently destroyed by Cysteine, as an essential amino acid for glutathione biosynthesis, benefiting from intracellular redox imbalance.

Apart from the photothermal cancer treatment, the photothermal cocrystal materials also display potential opportunities in antimicrobial therapy. Specifically, the PER‐TCNQ cocrystal with innovative NIR‐II absorption was prepared as nanoparticles via supramolecular assembly and nanoprecipitation, which was applied for photothermal antibacterial therapy in eliminating the Gram‐negative and Gram‐positive bacteria.^[^ ^55^ ^]^ The antibacterial activity of PER‐TCNQ NPs against *Escherichia coli* and *Staphylococcus aureus* was assessed by the number of colony‐forming units (CFU) on the agar plates. Both *Escherichia coli* and *Staphylococcus aureus* colonies on agar plates were divided into four groups: 1) Control; 2) NIR‐II irradiation; 3) PER‐TCNQ NPs; 4) PER‐TCNQ NPs + NIR‐II irradiation. Under the irritation of 1064 nm laser for 15 min, the relative viability toward *Escherichia coli* and *Staphylococcus aureus* may be reduced to 0 and 1%, respectively, at a concentration of 20 mg mL^−1^ in experimental group 4 (PER‐TCNQ NPs + NIR‐II irradiation) (Figure 7c). It indicates that PER‐TCNQ NPs with PTC properties could effectively eliminate *Escherichia coli* and *Staphylococcus aureus*. Subsequently, the morphological changes of bacteria in four different experimental groups after treatments were observed by scanning electron microscopy (SEM). As shown in Figure 7d, in experimental group 4 (PER‐TCNQ NPs + NIR‐II irradiation, 15 min), the surfaces of *Escherichia coli* and *Staphylococcus aureus* became rough, showing more intense damage. It illustrates that PER‐TCNQ NPs have a superior antibacterial ability through photothermal treatment. In conclusion, PER‐TCNQ NPs exhibit an excellent PTC ability for antibacterial therapy. Likewise, CT nanococrystal (TAPP‐TCNQ NPs) of 5,10,15,20‐tetra(4‐aminophenyl) porphyrin (TAPP) and 7,7,8,8‐tetracyanoquinolinedimethane (TCNQ) was demonstrated by Xie and co‐workers.^[^ ^61^ ^]^ Significantly, TAPP‐TCNQ NPs display great photothermal and photodynamic properties for efficiently eliminating bacteria both in vitro and in vivo with an 808 nm laser irradiation. In vitro experiments, TAPP‐TCNQ NPs could availably kill both *Escherichia coli* and *Staphylococcus aureus* with an antibacterial efficiency of up to over 95% under an 808 nm laser irradiation. Furthermore, a wound infection model of mice was established to explore the antibacterial capacity of TAPP‐TCNQ NPs in vivo. Under 808 nm laser irradiation, the amounts of the bacteria in the wound with the prepared TAPP‐TCNQ NPs would be significantly reduced, attributing to a great photothermal and photodynamic effect. After a while, the tissues treated with both laser and TAPP‐TCNQ NPs displayed a significant reepithelialization and more new blood capillaries. It suggests that TAPP‐TCNQ NPs can effectively extinguish bacteria in vivo and accelerate the healing of infected wounds under irradiation with an 808 nm laser. Therefore, TAPP‐TCNQ NPs are applied in antibacterial therapy with an excellent antibacterial effect both in vitro and in vivo. In short, the organic CT cocrystal strategy could significantly provide great responses in the NIR bio‐window for biotherapy, which dramatically facilitates the PTC cocrystal materials to be applied in the tumor treatment and antimicrobial therapy, etc.

### 3.3 Seawater Desalination

Solar‐driven water evaporation, which uses solar energy as a sustainable energy source, is a promising way to provide clean water scarcity solutions with minimal environmental impact.^[^ ^78^ ^]^ However, the low solar energy absorption and high heat loss of conventional volumetric water heating systems lead to a low PTC efficiency, which hinders their practical application in seawater desalination.^[^ ^79^ ^]^ The rational design of both NIR photothermal materials as efficient solar energy capturers, and the novel evaporation systems that utilize the interfacial heating concept, supplied a facile and feasible strategy to overcome the defects of traditional evaporation systems and improve the efficiency of water evaporation.^[^ ^80^ ^]^ Recently, cocrystal materials with fascinating PTC were used as ideal materials for solar‐driven water evaporation, which is conducive to their light absorption of the entire solar spectrum. Therefore, it is greatly significant to explore the interfacial heating system, which consists of cocrystal materials with NIR photothermal properties and interfacial materials.

**Figure 8:**
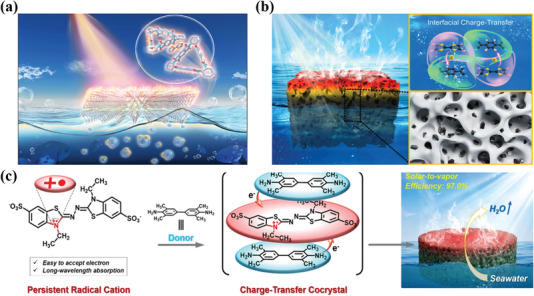
The various interface materials based on photothermal cocrystals for seawater desalination. a) Interface materials of PTFE.^[^ ^26^ ^]^ Copyright 2020, Royal Society of Chemistry. b) polydimethylsiloxane. Reproduced with permission.^[^ ^34^ ^]^ Copyright 2020, American Chemical Society, and polyurethane. c) Combined with cocrystals for solar‐driven water evaporation. Reproduced with permission.^[^ ^42^ ^]^ Copyright 2022, Wiley‐VCH.

Zhao and co‐workers first combined a CT cocrystal based on TTF and Tri‐PMDI with interfacial materials of polytetrafluoroethylene (PTFE) membrane to obtain a solar‐driven water evaporation system (**Figure** **8**a).^[^ ^26^ ^]^ Due to the TTF‐Tri‐PMDI with the unique quasi‐2D structure, the increased specific surface area facilitates effective sunlight absorption. Specifically, interfacial materials of PTFE membrane with both low density and multiple apertures are favorable for the effective location of TTF‐Tri‐PMDI materials. It is also useful to be self‐floated on the water to construct a solar‐driven water evaporation system of TTF‐Tri‐PMDI‐PTFE membrane with an evaporation efficiency of 46.2%. Besides, a high‐efficiency solar absorber using organic CT cocrystal was demonstrated by Lee and co‐workers.^[^ ^34^ ^]^ This excellent water evaporation system with an evaporation efficiency of 90.3% is constructed by interfacial material of polydimethylsiloxane (PDMS), a porous polymer scaffold, and a TTF‐TCNQ cocrystal with full solar spectrum absorption (Figure 8b). Notably, the efficient water evaporation capacity of the solar absorber is mainly from two factors. On the one hand, the PMDS scaffold can load effectively with the TTF‐TCNQ because of its rich porous microstructure. On the other hand, broad‐spectrum absorption of TTF‐TCNQ originated from intense intermolecular CT interaction. An excellent water evaporation system with an evaporation rate of up to 1.67 kg m^−2^ h^−1^ through 90.3% solar conversion under 1 Sun is applied in practical multiple purifications of wastewater. Except for the PTFE membrane and the PMDS scaffold, Chen and co‐workers also proposed a PU foam as interfacial material, loading CT cocrystal to establish an effective interfacial water evaporation system (Figure 8c).^[^ ^42^ ^]^ Interestingly, its unique CT cocrystal was prepared by TMB with intense electron‐donating and great persistency and electron affinity of ABTS^+•^, which imparted a great degree of electron delocalization between TMB and ABTS^+•^. Combined with the inherent long‐wave absorption properties of ABTS^+•^, the self‐assembled TMB‐ABTS^+•^ cocrystal could effectively capture the entire solar spectrum and demonstrate significant PTC ability. Furthermore, PU foam possesses a large number of pores and capillaries. It indicates that this prepared PU foam could effectively transport the water to the interface and facilitate the process of evaporation. Therefore, these prepared TMB‐ABTS^+•^‐PU composite foams with an interfacial water evaporation system demonstrated an outstanding water evaporation rate of 1.407 kg m^−2^ h^−1^ and a significant solar‐driven water evaporation efficiency of 97.0% under 1 solar irradiation. Considering the intense CT interaction between donors and acceptors, cocrystal materials can obtain a property of entire solar spectrum absorption, which can be used as an excellent candidate material for seawater desalination applications.

### 3.4 Other Applications

**Figure 9:**
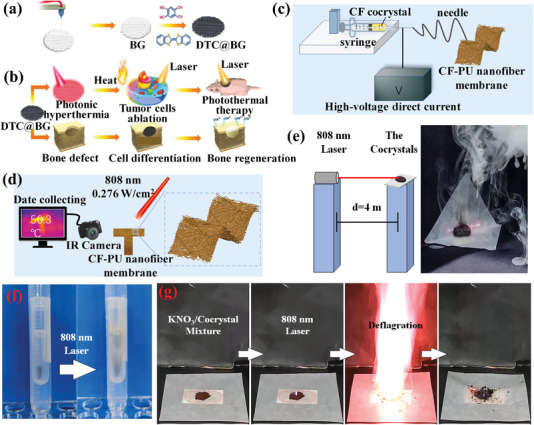
The photothermal cocrystals for the application of 3D‐printing technique, electrospinning technology, and laser ignition. a) Preparation of bifunctional DTC@ bioactive glass (BG) scaffolds via a 3D‐printing technique. b) The procedure of DTC@BG scaffolds applied in eliminating bone tumors via photothermal and accelerating the newborn bone growth. Reproduced with permission.^[^ ^80^ ^]^ Copyright 2022, Wiley‐VCH. c) CF‐PU nanofiber film was gained via electrospinning technology. d) CF‐PU nanofiber film was applied in photothermal imaging. Reproduced with permission.^[^ ^58^ ^]^ Copyright 2022, American Chemical Society. e) Remote trigger experiment setup and photo of successful TMPD‐TBBQ triggering in this setting; f) pictures of TMPD‐TBBQ before and after 808 nm laser irradiation after encapsulation; g) deflagration experiments with a handheld 808 nm laser on a mixture of KNO_3_ and TMPD‐TBBQ powders. Reproduced with permission.^[^ ^37^ ^]^ Copyright 2022, American Chemical Society.

In order to take the PTC cocrystal for the application of fabricating artificial bones as an example, Chen and co‐workers designed a bifunctional therapeutic biomaterial platform DTC@BG scaffold for the photothermal elimination of osteosarcoma and accelerated regeneration of bone defects.^[^ ^81^ ^]^ Depended on DTC cocrystal madding up of DBTTF and TCNB, the DTC@BG scaffold was constructed by a 3D‐printing bioactive glass (BG) scaffold as verified in **Figure** **9**a, which process a NIR PTC efficiency of 26.8%. Remarkably, the DTC cocrystal exhibits a narrow HOMO–LUMO gap of 1.3 eV, which agrees with the experimental absorption spectrum of 904 nm. Accordingly, the DTC cocrystal demonstrates a high PTC efficiency of 21.9% under irradiation with 808 nm. Interestingly, the DTC cocrystal was exquisitely introduced into the BG scaffold with the ability to reconstruct new bone via 3D‐printing to obtain DTC@BG scaffold, which could enhance its NIR PTC efficiencies. It illustrates that DTC@BG scaffold presents excellent potential in photothermal therapy. Furthermore, the DTC@BG scaffold is applied in the treatment of bone tumors to effectively eliminate the bone tumor and rapidly facilitate the reconstruction of new bone (Figure 9b). As for eliminating the bone tumor, the apoptosis rate of osteoma cells is up to 72.7% in vitro under the effect of DTC@BG scaffolds and 808 nm laser. It confirms that the DTC@BG scaffold could effectively ablate tumor cells in vitro. Furthermore, the tumors of the mice treated with the DTC@BG scaffold and 808 nm laser exposure were completely eradicated throughout the observation period without further recurrence, suggesting an inhibited regrowth of bone tumors by the DTC@BG scaffold in vivo. Notably, the ability of PTC therapy was attributed to DTC with NIR PTC properties. Thus, the DTC@BG scaffold could generate heat under laser irradiation to effectively eliminate the tumor cells in PT therapy. Besides, as for the capability of osteogenesis after implantation of the DTC@BG scaffold for eight weeks, the percentage of bone volume at bone defect sites in mice is up to 43.5%, as well as the bone mineral density of defect sites reaches 4.8 g m^−3^. It elucidates that the DTC@BG scaffold facilitates the new bone formation and defect healing. Significantly, the characteristic of bone defect regeneration is mainly derived from two sources. On the one hand, DTC cocrystals could enhance the surface roughness of human bone mesenchymal stem cells, facilitating the adherence and proliferation of human bone mesenchymal stem cells.^[^ ^82^ ^]^ On the other hand, the sodium (S) element in the cocrystals may promote protein absorption through interactions between S and protein, thereby promoting osteoblast proliferation and differentiation.^[^ ^83^ ^]^ Therefore, when the DTC@BG scaffolds were inserted into the bone defects, it could promote the proliferation differentiation of bone tissues, and the newborn bones were generated in the defect regions at the end (Figure 9b). Similarly, Zhuo and co‐workers recently reported a lager‐area photothermal nanofiber membrane based on CT cocrystal and PU for photothermal imaging^[^ ^58^ ^]^ by electrospinning technology. The process of fabricating CF‐PU nanofibrous materials mainly involves the injection of coronene‐F_4_TCNQ (CF) cocrystal based on coronene and F_4_TCNQ and the molding of fibrous materials (Figure 9c). Notably, under irradiation with an 808 nm laser, the temperature of CF‐PU nanofibrous materials increase from 26 °C to 52 °C in 250 s, which possesses an excellent NIR PTC efficiency of 53.7%. Therefore, the CF‐PU nanofiber membrane has significant potential in NIR photothermal imaging. As expected, CF‐PU nanofiber films were cut into a T shape. Then the pattern T becomes brighter as the increased irradiation time and power density of NIR light, which provides a novel method to rationally design large‐area photothermal membranes for photothermal imaging (Figure 9d).

Meanwhile, our group prepared a CT cocrystal based on TMPD and TCBQ via a self‐assembly method, which displays the performance of high temperatures for a novel application of laser ignition under NIR laser irradiation.^[^ ^37^ ^]^ As shown in Figure 9e, these prepared TMPD‐TCBQ cocrystal materials could easily be triggered remotely at distances of up to 4 m. Furthermore, the superior penetration ability of the NIR laser allowed TMPD‐TBBQ encapsulated in a three‐layer polypropylene tube to be successfully triggered under an 808 nm laser (0.6 W) from the outside (Figure 9f). It suggests that the exotherm of the TMPD‐TCBQ cocrystal could be triggered when these CT cocrystal materials were encapsulated in transparent packaging. To show the laser ignition performance of TMPD‐TCBQ, the mixture powder consisted of KNO_3_, a NIR‐laser‐unresponsive oxidant, and TMPD‐TCBQ was deflagrated drastically after two seconds of 808 nm laser irradiation as illustrated in Figure 9g, which demonstrate unprecedented advantages as a low‐power NIR laser‐responsive initiator. In a word, cocrystal strategy could regulate their absorption via the self‐assembly of donors and acceptors to obtain PTC materials of particular wavelength for different PTC applications, which meets the ever‐growing demand for PTC applications.

## 4 Conclusion and Outlook

In conclusion, this review firstly demonstrated a comprehensive overview of organic CT cocrystal photothermal materials from their photothermal‐conversion mechanism to photothermal effects with significant ability in various applications. First, we proposed that organic cocrystal photothermal materials with various frameworks of raw materials demonstrated different performances of PTC, which originated from the different abilities of both light‐harvesting and nonradiative decay processes. Then, we found that some specific structures in raw materials could improve the PTC capability of cocrystals by facilitating nonradiative decay processes. For example, the high‐efficiency PTC of the organic cocrystals material consisting of an acceptor TCNQ is attributed to the free rotation of the —C(C≡N)_2_ group of TCNQ, which is conducive to the effective nonradiative decay through the VR and IC. Moreover, PTC cocrystals with abundant free ions could generate substantial space charge transfer, promoting light absorption in the entire solar spectrum for a significant PTC ability. Furthermore, PTC cocrystal without thermostability could produce a dramatic exothermic heat under NIR laser irradiation, which is due to the polymerization reaction. According to their different photophysical properties, organic PTC cocrystals were applied in various applications. Specifically, the large specific surface area of organic PTC cocrystals was useful for the photothermal imaging application.

Additionally, PTC cocrystals also possess excellent NIR absorption and low biological toxicity, showing a promising application in biological therapies, which can merge a photothermal image into photothermal therapies to achieve the integration of diagnosis and treatment. Furthermore, PTC cocrystals with a broad absorption in the entire solar spectrum would combine with excellent interface materials to obtain effective interface water evaporation systems for seawater desalination. Except for application in seawater desalination, PTC cocrystals with a full solar spectrum absorption may be used in photothermal to thermoelectric conversion, which can provide a novel insight to generate electricity in an environmentally friendly path. Remarkably, PTC cocrystals without thermostability under laser irradiation could be used for ignition materials, such as being applied in gunpowder to the initiator. Of course, PTC organic cocrystals can be also applied in the fields of thermally conductive materials, heat storage materials, and so on in the future.

There is no denying that the organic photothermal cocrystal is thriving and promising. However, as an emerging field of research, some challenges still need to be solved for the practical application of organic cocrystal photothermal materials. First, the systems of organic PTC cocrystal materials that have been reported recently are not abundant. At present, the PTC performance of these organic cocrystal materials is mainly concentrated in the NIR region, while the cocrystal materials with the PTC ability of mid‐infrared and even far‐infrared are rarely reported. Moreover, since organic PTC cocrystal materials are in a gradual development process, the photothermal mechanism of materials is unclear, resulting in that the efficiency of organic PTC cocrystal obtained is less than 100%, which attribute to the competition between radiative and nonradiative transitions in the energy dissipation mode. Therefore, it is necessary to develop a novel strategy to remove the radiative transitions in the energy dissipation mode, which achieves the PTC efficiency is up to 100%. Finally, the application of organic PTC cocrystal materials is still relatively narrow at present. It is necessary to develop more novel applications according to both the photophysical properties and photothermal characteristics of materials, such as photothermal‐thermoelectric conversion, laser ice‐breaking, and so on. In a word, the organic cocrystal strategy will ultimately achieve an integrated design from the molecular structure to their material function, which significantly facilitates the development of photothermal materials in diverse fields.

## Conflict of Interest

The authors declare no conflict of interest.

## Author Contributions

Y.‐T.C. and M.‐P.Z. contributed equally to this work. X.‐Y.W. and W.‐B.C. consulted and collected relevant literatures. Y.‐T.C. and M.‐P.Z., K.‐Q.Z., and M.‐D.L. discussed the interpretation of results and wrote the paper. All authors discussed the results and commented on the manuscript.
